# Keep focussing: striatal dopamine multiple functions resolved in a single mechanism tested in a simulated humanoid robot

**DOI:** 10.3389/fpsyg.2014.00124

**Published:** 2014-02-21

**Authors:** Vincenzo G. Fiore, Valerio Sperati, Francesco Mannella, Marco Mirolli, Kevin Gurney, Karl Friston, Raymond J. Dolan, Gianluca Baldassarre

**Affiliations:** ^1^Wellcome Trust Centre for Neuroimaging, Institute of Neurology, University College LondonLondon, UK; ^2^Laboratory of Computational Embodied Neuroscience, CNR, Istituto di Scienze e Tecnologie della CognizioneRoma, Italy; ^3^Adaptive Behaviour Research Group, Department of Psychology, University of SheffieldSheffield, UK

**Keywords:** basal ganglia, dopamine, selection, novelty, iCub, intrinsic motivation

## Abstract

The effects of striatal dopamine (DA) on behavior have been widely investigated over the past decades, with “phasic” burst firings considered as the key expression of a reward prediction error responsible for reinforcement learning. Less well studied is “tonic” DA, where putative functions include the idea that it is a regulator of vigor, incentive salience, disposition to exert an effort and a modulator of approach strategies. We present a model combining tonic and phasic DA to show how different outflows triggered by either intrinsically or extrinsically motivating stimuli dynamically affect the basal ganglia by impacting on a selection process this system performs on its cortical input. The model, which has been tested on the simulated humanoid robot iCub interacting with a mechatronic board, shows the putative functions ascribed to DA emerging from the combination of a standard computational mechanism coupled to a differential sensitivity to the presence of DA across the striatum.

## 1. Introduction

Distinct functions are ascribed to striatal dopamine (DA) in relation to the type of outflow (tonic/phasic) expressed by this neuromodulator and the experimental context. “Tonic” DA release is caused by the removal of inhibitory constraints affecting spontaneously active DAergic neurons (Floresco et al., 2003; Grace et al., 2007). This low frequency mode of DA activation is considered as encoding average rewards (Niv et al., 2007; Beierholm et al., 2013), the presence of stressors (Cabib and Puglisi-Allegra, 2012) or novel stimuli (Lisman and Grace, 2005), and more recently as an indicator of precision of prior beliefs (Friston et al., 2012). As far as its function, tonic DA is mainly investigated for its effects on motor control: one influential account posits a role in mediating the vigor with which a subject pursues desired outcomes (Niv et al., 2007) which might be limited to approach strategies (Guitart-Masip et al., 2012). This overlaps with a proposed role in mediating the disposition to exert and sustain effort in pursuing a goal (Salamone et al., 2003; Salamone and Correa, 2012) and incentive salience in motivation or “wanting” (Berridge and Robinson, 1998; Peciña et al., 2003). Recent human evidence has also suggested a role attaining a balance between model free and model-based behaviors (Wunderlich et al., 2012), a formulation consistent with models of habitual versus goal control in Parkinson disease (Redgrave et al., 2010) and with DA's established role in reasoning, cognitive flexibility, planning, and working memory (Montague et al., 2004; Cools and D'Esposito, 2011).

Phasic DA release results from a direct glutamatergic excitation of DAergic neurons (Floresco et al., 2003). There is substantial agreement these short burst firings play a key role in triggering learning processes, but the exact information they convey is disputed. The main proposal is that DA bursts report a reward prediction error resulting in reinforcement learning, a key element in behavior that leads to reward maximization (Sutton and Barto, 1998; Schultz, 2007). However, phasic DA is also considered as implicated in signaling saliency (Redgrave et al., 1999b) and agency-related novelty (Redgrave and Gurney, 2006; Redgrave et al., 2008).

Whether DA is considered as signaling the presence of unexpected or novel stimuli and independently of their association with the agent's actions or priors, there exists a strong relation between DA and the broad category of intrinsically motivating stimuli. These are motivations guiding learning in the absence of primary “extrinsic” rewards such as food, water, and pain, and are directed to acquire knowledge and skills exploitable in later stages (Ryan and Deci, 2000; Baldassarre and Mirolli, 2013; Mirolli et al., 2013). The key feature of these motivations relies in the optimization of the information flow (Tishby and Polani, 2011), narrowing the amount of information that needs to be processed and motivating risky, but potentially fruitful, explorations in a changing environment (Kakade and Dayan, 2002; Ranganath and Rainer, 2003; Kaplan and Oudeyer, 2007; Düzel et al., 2010).

DAergic neurons are localized in a restricted brain region mainly the ventral tegmental area (VTA) and substantia nigra pars compacta (SNpc). By contrast, its targets, including the striatal region, are broad and heterogeneous. This is often seen as suggesting that DA cannot encode fine grain information and this lack of target specificity hints that its effects may be the expression of a coarse influence (Schultz, 2007). Among DA principal projection targets is the striatum, a component in a complex circuitry involving the substantia nigra pars reticulata (SNr), the globus pallidus (GP) and the sub-thalamic nucleus (STN) that together form the basal ganglia. These nuclei are connected to the cortex via the thalamus to create parallel reentrant loops, where motor, associative, and ventral (limbic) cortices project to their specific target compartments in the striatum—respectively putamen (Put), caudate (Cau), and nucleus accumbens (NAcc)—(Alexander et al., 1986; Haber et al., 2000; Utter and Basso, 2008; Miyachi, 2009). With minor exceptions, these loops show qualitatively similar internal structure across functional areas (Nakano, 2000; Redgrave et al., 2010). The features characterizing this circuitry have led researches to ascribe two functions to the basal ganglia: first, as responsible for action selection modulated by tonic DA outflow (Redgrave et al., 1999a), and, second, as mediator of reinforcement learning triggered by phasic DA via instrumental conditioning and novelty detection (Schultz, 2006). Thus, current theories highlight a neuromodulatory gain control and action selection role for DA or, alternatively, focus on its role in mediating the synaptic plasticity that underlies learning. Our approach rests upon coupling these two roles so that action selection and learning become an integral part of learning how to select actions. We will see later that this involves a closed causal chain involving the dopaminergic modulation of cortical plasticity and the cortical drive of phasic and tonic DAergic responses.

The core of our proposal is a new integrated hypothesis of the interaction between DA and cortical-striatal circuitry. In particular, we propose that DA's putative functions result from the combination of a differential sensitivity characterizing striatal subregions and the ability of DA to dynamically modulate a competition taking place within different basal ganglia nuclei. The present models show how DA affects the gain of a striato-cortical loop, altering the range of inputs capable of triggering a selection, the time required to perform a selection, and the ability of the system to persevere in a selection despite changes in the input. This mechanism is coupled with a differential sensitivity each part of the striatum exhibits to DA levels. This hypothesis is consistent with data describing the distribution of DA receptors in the striatum (Beckstead et al., 1988; Piggott et al., 1999) and it enables the agent to switch between behavioral strategies depending on the type of motivating stimuli perceived.

To support our hypothesis, we first simulate the activity of a single striato-cortical loop providing it an external arbitrary input and recording the way its processes are modified by the different outflows of DA. Secondly, we present a more complex model grounded on three striato-cortical loops, interconnected via the cortex, respectively for the control/selection of: arm actions (Put and pre-motor cortex, PMC), attention/associative processes for the selection of eye gaze (Cau and frontal eye field, FEF), and executive control for goal-directed behavior (NAcc and prefrontal cortex, PFC).

Both models are used in a series of simulated embodied tests performed on the humanoid robot iCub (Metta et al., 2010). The single loop model shows how increasing DA outflow enhances the probability of performing any selection (akin to action vigor) and leads to an increased perseverance of the selection in the face of distractors and variable information from environment. The three-looped model is used to solve a task requiring sensory-driven and novelty-driven exploration of a device having buttons and lights (the *mechatronic board*, cf. Taffoni et al., 2013): the agent is required to learn via intrinsic motivation (i.e., unexpected visual stimuli, Reed et al., 1996) and to exploit the acquired associations when extrinsic rewards appear in the environment. The next section will describe the details of the parts of the basal ganglia we have focussed on, neglecting others to simplify the overall complexity of the biological system the models refers to. Despite these simplifications, we think the results of the tests show that the DA-based mechanisms illustrated above can play several important adaptive functions such as the guidance of sensory- and novelty-driven exploration, the exploitation of goal-directed (model-based) action-outcome associations, and the saving of energy (rest) when no motivating stimuli are perceived.

## 2. Materials and methods

### 2.1. Basal ganglia: anatomy and circuitry

The multifunctional role ascribed to the action of DA within the striatum renders it unsurprising that the basal ganglia are themselves implicated in guiding perception, attention, learning, and memory processes, beside motor control. Both empirical evidence (Mink, 1996; Redgrave et al., 1999a; Grillner et al., 2005; Hikosaka, 2007) and computational modeling (see Humphries et al., 2006; Prescott et al., 2006; Baldassarre et al., 2012; Humphries et al., 2012, for the most closely related to the present model and Frank, 2011 for a general review) converge on the idea that a core element of basal ganglia function involves removal of tonic inhibition so as to realize a selection of its input.

**Figure 1:**
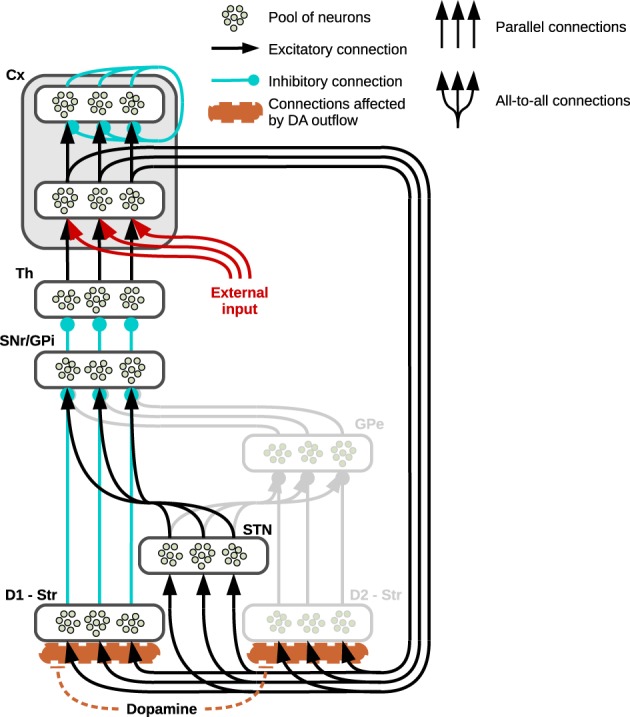
**Prototypical striatocortical loop used in simulations**. The neural structure shows three “channels” to exemplify its connectivity: the pools of neurons belonging to different neural subregions of the basal ganglia are connected either via localized (parallel) or diffuse (all to all) connections. The cortex (Cx) is divided into two neural layers, the inner layer is part of the striatocortical loop, receiving its incoming signal from the thalamus (Th) and propagating it to the striatum (Str) and the sub-thalamic nucleus (STN) whilst the external layer functions as the output of the system. In the single loop test showing the effects generated by arbitrary DA outflows, an external input reaches the inner layer of the cortex (here represented in red). The striatum is divided into two areas: the direct pathway involves the area of striatum characterized by the presence of D1 receptors (D1—Str) which is connected either to the Substantia Nigra Pars Reticulata (SNr) or the internal Globus Pallidus (GPi). The indirect pathway (represented in gray because it is not part of the present simulations) involves the part of striatum mainly characterized by D2 (D2—Str) and the external Globus Pallidus (GPe).

The basal ganglia receive massive input from most regions of cortex and provide a processed output to the thalamus, which closes the loop via reconnection back to the cortex. The circuitry characterizing the cortico-thalamic connection is also rather complex: the thalamus reaches layer IV of the cortex and this reaches the striatum via layers III and V whilst another loop involving directly thalamus and cortex is closed via layer VI (Douglas and Martin, 2004; da Costa and Martin, 2010). For the purpose of this study, the architecture will capture only the features characterizing specific parts of the basal ganglia relevant to the objectives of this work, leaving aside the complex interaction involving the other two main actors in this loop, namely cortex and thalamus (see section 4 for further details). One of these essential features is illustrated in Figure 1, which shows the parallel “channels” of neural populations characterizing a striatocortical loop (Alexander et al., 1986; Alexander and Crutcher, 1990; Gurney et al., 2001a,b): the striatum receives its localized input directly from the cortex and it propagates this signal via two distinct pathways, each originating in a subregion characterized by the presence of specific DA receptors. The first of these two striatal subregions shows a higher concentration of D1 receptors (having excitatory effect) and directly connects to the SNr (when considering the NAcc) and the internal part of the GP (Gpi, when considering the Cau and Put), forming the so-called direct pathway; the second subregion is characterized by greater concentration of D2 receptors (having an inhibitory effect) and its signals reach the SNr/GPi via a double inhibition involving the external Globus Pallidum (GPe), the so-called indirect pathway. Finally, a cortical input also reaches the STN which is connected directly to the SNr and GP via diffuse excitatory connections referred to as the hyperdirect pathway.

Parallel inhibitory channels of neural populations run through the whole loop, in both the direct and indirect pathways, as opposed to the diffuse excitatory connections between STN and SNr/GP. This structure results in a functional double competition between two regions preserving segregated activations and the region providing a diffuse undifferentiated signal: the former regions convey information about the values of each separate component of the input, whereas the latter conveys non-specific information about the general intensity of the incoming stimuli as a whole (Frank, 2006; Frank et al., 2007).

Assuming the input provided by the cortex already encodes the value or salience of the stimuli (Samejima et al., 2005; Lau and Glimcher, 2008; Kimchi and Laubach, 2009; FitzGerald et al., 2012; Znamenskiy and Zador, 2013), the input nuclei of the three pathways receive and process these saliencies in a continuous self-feeding process mediated by the presence of a closed loop: depending on the relative strength of activity in these pathways, the basal ganglia eventually alter these values preserving, increasing or suppressing the differences encoded. This process is mediated by the tonic inhibitory activity of the SNr/GPi—the output nucleus of the basal ganglia—whose channels can be selectively inhibited so as to release the corresponding population of neurons in the thalamus and resulting in a gating effect (Chevalier and Deniau, 1990; Gurney et al., 2001a,b). Most of this tonic activity is provided by the hyperdirect pathway which therefore concurs in reducing the chances that any of the channels in the SNr might be inhibited; on the contrary, the direct and indirect pathways compete in establishing which of the SNr/GPi channel has to be inhibited, the former favoring the strongest cortical inputs whereas the latter favors the weakest.

In the present study we are mainly interested in testing the effects on behavior due to an increase in DA outflows. Thus, we have simplified the structure of basal ganglia by relying on a model that focusses on the competition implemented by direct and hyperdirect pathways alone (Figure 1 shows the regions whose activity has not been simulated in light gray). This simplification is justified assuming that, due to the presence of the D2 receptors, increasing DA release causes the indirect pathway to decrease its activity, therefore—in a computational perspective—it diminishes its effect on the whole system, allowing the D1-related direct pathway to have a major role in the selections (Humphries et al., 2012). This choice is also consistent with data and models identifying indirect pathway structures as responsible for “No-Go” that is negatively correlated with increases of DA release due to high concentrations of D2 receptors (Frank et al., 2004; Surmeier et al., 2007; Frank, 2011; Guitart-Masip et al., 2012): the present models rely on a simplified structure which can be considered nonetheless accurate in analyzing most of the behaviors connected with high DA outflows and “Go” choices.

### 2.2. The computational model

The neural systems used for both simulation and embodied tests were developed with C++ libraries: these were tested for the first time in Baldassarre et al. (2012) and have been modified to deal with the new requirements concerning the neural architecture and the mechanics involving the simulated DA. The basic building block of the models is a *leaky integrator unit* defined by a continuous-time differential equation that simulates mean activity of a whole neural area or pool of neurons. This is a standard tool in firing rate models (Dayan and Abbott, 2005), modified to include the effects of the DA neuromodulation as follows:

(1) $$\tau_{g}{\dot{u}}_{j}=-u_{j}+b_{j}+(\epsilon +\lambda \;d)\;\Sigma_{i}w_{ji}y_{i}$$

where τ_*g*_ is a time constant (related to the nucleus or group, *g*, of units to which *j* belongs), *u*_*j*_ is the activation potential of unit *j*, *b*_*j*_ is the basal activation of such unit (if any), *w*_*ji*_ represents the connection weight between input unit *i* and unit *j*, and *y*_*i*_ the activation of input unit *i*.

**Figure 2:**
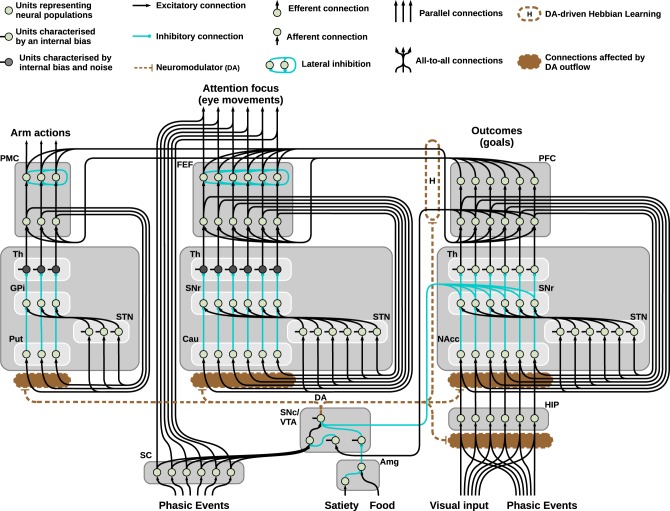
**Neural architecture of the model used to solve the mechatronic board task: three striatocortical loops reciprocally connected via learned cortico-cortical connections**. The system allows the agent to autonomously explore the environment, perceive intrinsically motivating signals (flashes of light), learn and exploit the learned association to pursue a maximizations of rewards.

To include the DAergic modulation, we assume DA enhancement of the signal reaching a target area can be simulated via a multiplicative effect: this is a standard computational strategy in simulating D1 specific effects (Fellous and Linster, 1998; Durstewitz, 2009) and is realized through the parameter *d*, representing the amount of DA released, and the coefficients ϵ and λ, respectively for the strength of the input independent of the presence of DA and the multiplicative effect DA exerts on the same input. These two coefficients have been set to ϵ = 1 and λ = 0 for all the units which are not affected by the DA release in the simulations, and 0 < ϵ < 1 and λ > 0 for the remaining units: besides the striatum, the three-looped model (see Figure 2) also shows the hippocampal simulated layer as being affected by the presence of DA in the way described here.

Equation (1) describes the activation potential of the units in the neural models where Equation (2) is a positive saturation transfer function defining the final activation of these units: the activity of all units here described is simulated relying on these two equations. The transfer function is defined as follows:

(2) $$y_{j}={\left[\tanh\left(\alpha_{g}\left(u_{j}-\theta_{g}\right)\right)\right]}^{+}$$

where tanh(.) is the hyperbolic tangent function, α*g* is a constant defining the slope of the hyperbolic function (per group), θ_*g*_ is a threshold parameter (per group) and [.]^+^ is a function defined as [*x*]^+^ = 0 if *x* ≤ 0 and [*x*]^+^ = *x* if *x* > 0. Notice that 0 < *y*_*j*_ < 1 for all *j*. Aside from the layers simulating the activity of the cortex, the threshold is always set to θ = 0: the cortical transfer function has been thresholded so that units activations are zero unless their corresponding activation potential exceeds its layer specific threshold (in the single loop simulation, these are set to 0.6 for the inner cortical units and 0.8 for external cortical units).

Finally, the three-looped model shown in Figure 2 consists of three separated loops for manipulation, attention, and executive control: respectively the dorsolateral, dorsomedial, and ventral striatocortical loop. During the task, these systems establish cortico-cortical connections that reciprocally bias the selection performed thanks to a Hebbian learning process guided by the presence of phasic DA. The equation describing this learning is as follows:

(3) $$\Delta w_{ji}=\eta_{\text{ctx}}\;g_{j}y_{i}\;({\hat{w}}_{\text{ctx}}-w_{ji})\;{\left[d-\zeta \right]}^{+}\;$$

where *y*_*i*_ and *g*_*j*_ represent the activities of the connected units (belonging respectively to the cortical external layer in the pre-synaptic loop and the cortical inner layer in the post-synaptic loop), *w*_*ji*_ is the connection weight between *y*_*i*_ and *g*_*j*_, η_ctx_ is a learning rate and *ŵ*_ctx_ is a maximum value reachable by *w*_*ji*_. The neuromodulator is here thresholded: [*d* − ζ]^+^ represents the amount of DA required to overcome a threshold ζ, where [.]^+^ is defined as in Equation (2). This threshold is set higher than any tonic outflow variation, therefore allowing learning processes only in presence of high peaks of DA corresponding to phasic activations.

The DA bursts required for LTP Hebbian learning are triggered by sudden luminance variances perceived by the system/agent via the superior colliculus (SC): this region provides fast and strong signals to the DAergic units, which result in the simulated DA bursts (resembling the actual connectivity and function as described by Redgrave and Gurney, 2006). Both tonic and phasic DA releases are simulated with one component representing the overall activity of both the VTA and the SNc: the activity of the single DAergic unit is controlled by both excitatory and (tonically active) inhibitory units.

In order to simulate the presence of an agency-related predictor, the same learning process expressed in Equation (3) is also used to establish excitatory connections between the inner cortical layer of the simulated PFC, part of the ventral loop, and an interneuron unit in the DAergic area: this direct connection simplifies the actual pathway responsible for this signal control functioning, which may involve the lateral habenula (Hikosaka et al., 2008). This learning process, triggered by the presence of DA bursts, eventually leads to suppression of phasic DA responses: the agent relies on the acquired cortico-cortical associations between specific combination of attentional/motor selections and PFC activity triggered by the perception of motivating stimuli to provide the ventral loop with the required information about the proximal cause of any experienced motivating stimuli. Since motor and attentional selections temporally precede the stimulus, once the association is learnt, this information is sufficient to cause activation of an inhibitory unit in the DAergic area (via PFC) before the actual stimulus takes place. As a result, an action causing unexpected changes in the environment, such as luminance variance in the present task, will trigger DA bursts that engage learning processes among cortical regions and between the PFC and the DAergic area. However, if manages to successfully repeat the correct action on the proper target, the resulting change in the environment will eventually become predicted, therefore preventing an input coming from the SC from triggering any more DA bursts.

The learned cortico-cortical connections among different striato-cortical loops are instances of inverse and forward models (Gurney et al., 2013). Inverse models implement here the links between goal representations and action representations, important for the recall of actions on the basis of the pursued goals in goal-directed behavior. The forward models, instead, allow the anticipation of the accomplishment of a certain outcome when a certain action is performed.

This role of DA in the self-assembly or bootstrapping of intrinsically valuable sensorimotor sequences is reminiscent of early simulations of value-dependent learning using neuronally plausible models (Friston et al., 1994). In brief, the dopaminergic reinforcement of stimulus-response and response-stimulus links by DA depends upon phasic dopaminergic discharges. By introducing dopaminergic plasticity into the cortical projections eliciting these discharges, one introduces a circular causality, in which innately or intrinsically rewarding stimuli transfer their value to their sensory or motor precedents. This form of learning has formal links with actor-critic models in reinforcement learning, accounts for the transfer of phasic dopaminergic responses from unconditioned to conditioned stimuli and provides a physiologically grounded account of how sequences of exploratory or exploitative behavior emerge.

Among the remaining components of the model pictured in Figure 2, the hippocampus (HIP) is composed by a single layer of units encoding spatial representations: the activity of these units slowly decreases as a response to the incoming input. The slow decrease of the input (which starts from the maximum value of 1 to reach its minimum value of 0.1 in roughly 2 min) is determined by the time of exposure to the visual stimulus: this process simulates habituation to novel stimuli, leading to high responses of HIP to novel stimuli located in space (as it happens during visual exploration of a new environment) and low responses in presence of familiar items.

The projections of the HIP via the NAcc and the SNr to DAergic areas drives changes toward a tonic response mode of the simulated DAergic unit, which itself affects the activity of the HIP thus creating a loop. This circuitry is consistent with HIP connectivity and functioning (Grace et al., 2007) as the literature describes it as one of the major systems responsible for novelty detection and the related regulation of tonic DA release (Lisman and Grace, 2005; Düzel et al., 2010). The HIP is not the only part of brain that responds to novelty and habituates (see Ranganath and Rainer, 2003, for a review). However, coherently with the choice illustrated above on the HIP as the only source of novelty detection in the model, we included in the model only HIP habituation. This assumption was sufficient to have a brain mechanism performing novelty detection and habituation, and the consequent novelty based tonic DA regulation.

The simulated DAergic area is thus controlled by activity of SC (causing phasic DA bursts), the PFC (inhibiting the signal coming from the SC and suppressing DA bursts), SNr (responsible for tonic inhibitory control mainly due to the HIP) and finally a simplified amygdala (Amg): this component affects the activity of a tonically active interneuron in the DAergic area, resulting in strong increase in the DA outflow when a reward is perceived (i.e., simulating the perceived presence of food in one of the boxes).

Regarding the BG-cortical structures, the manipulation striato-cortical loop is characterized by three channels and controls the arm in the robotic set-up allowing selection among three possible actions (one per channel). Both the attentional loop and the ventral loop have six channels: the first controls foveation among six possible locations in space and the second controls the selection of the desired outcome to pursue. The three loops are similar, showing differences in only a few key parameters: among these, it is important to stress the presence of random noise in the thalamic parts of the manipulation and attentional loops and the presence of a different value for the coefficient λ for each of the striatal layers. The noise, which is essential to perform random exploration, is smoothed using a leaky integrator (Equation 1) and therefore is controlled by two parameters, one for the strength of the input and one for the decay speed (see Table 1). The coefficient λ (Equation 1), on the other hand, simulates the differential sensitivity to the presence of DA characterizing different striatal regions. This differential sensitivity will be shown to be essential for endowing the system with a flexible behavioral expression and for avoiding multiple fixated selections.

**Table 1:** **Table of essential parameters marking the difference among the three loops and the learning processes: the complete set of parameters is available for download (see instructions in the Supplementary Material)**.

**Parameters**	**Attention**	**Arm action**	**Goal**
λ	2.5	1.5	1
θ_*g*_ layer 1	0.4	0.6	0.1
θ_*g*_ layer 2	0.8	0.8	0.6
Lateral inhibitions	2	0.2	0
Noise in Th	20	30	0
Noise decay	1000	2000	0
Learning processes		(ζ coefficient)	
Cortico-cortical		0.2	
Predictor		0.00008	

*The tuning has been carried out by comparison with behavioral results*.

The biological plausibility of this hypothesis is grounded on the known distribution of D1 receptors within the striatum: there is a gradient of D1 receptor density within each subregion, with the Cau and the NAcc having, respectively, the highest and the lowest concentrations (Beckstead et al., 1988; Piggott et al., 1999). Assuming a higher concentration of D1 makes a neural region more sensitive to any variation of DA outflow is consistent with the model computational requirements to solve the mechatronic board task. In the model, DA alters the gain in each of the three feedback loops, having in the attentional loop (involving the Cau) the most sensitive system, in the manipulation loop (involving the Put) mid sensitivity and in the executive control loop (involving the NAcc) the system that requires the most DA release to be activated.

### 2.3. Robotic setup and mechatronic board task

**Figure 3:**
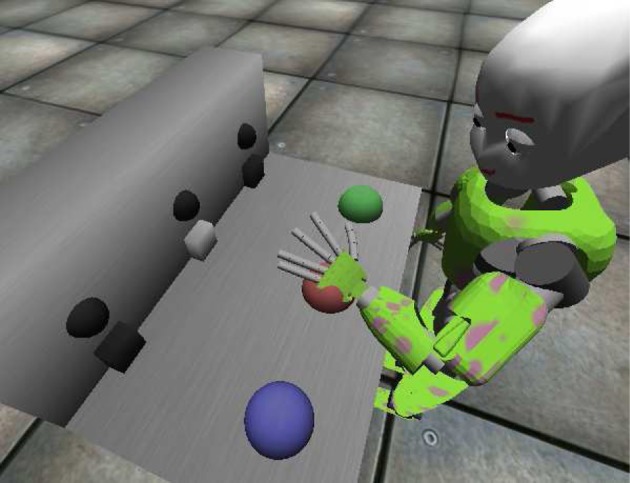
**The iCub in its simulated environment interacting with the mechatronic board**. The image is captured whilst the robot is pressing the red button, triggering the corresponding light.

The *iCub*^1^ is a humanoid robot whose dimensions resemble those of a 5 years old child. This robotic platform is characterized by an high number of degrees of freedom (16 for each arm, 5 for the head-eyes, 3 for the torso), so it is particularly fit to deal with tasks involving “human-like” movements. The official simulator of the *iCub* has been used to run the experiments concerning vigor and the solution to the *mechatronic board task* (see Figure 3). A mechatronic board, described in Taffoni et al. (2013), has been simulated and employed as the test environment. In order to match the requirements of the three-looped neural system here described, three actions (namely “grab,” “wipe,” and “press”), have been implemented to move the robot left hand in different ways and positions. Any selected action is always performed on the target the iCub is looking at. Through its movements the robot can interact with the mechatronic board, triggering light-flashes (lasting 1 s) when the proper action is performed on one of the correct targets: the time required to complete an action varies between 2 and 3 s circa (0.5 for a foveation), depending on the starting position of the arm and the final target. Note that, despite the name, the actions “grab” and “wipe” denote simply dummy actions, i.e., actions with no consequence on the board.

The control works in continuous time reflecting the activity of the neural system, so that both the actions and the targets can be changed or stopped at any time. This feature allows the experimenter to add and relocate a reward in any of the accessible locations at any time whilst the robot is interacting with the environment. A link to a short movie showing the robot interacting with the actual mechatronic board is provided in the Supplementary Material.

The task the agent is dealing with is rather simple: it requires exploration of an unknown environment, learning of agency-related associations due to the presence of intrinsically motivating stimuli (light flashes) and recall/exploitation to pursue the maximization of extrinsically motivating rewards. The mechatronic board consists of three buttons and three transparent boxes (see Figure 3): when the correct action is performed on any button (press), the box opens and the associated light flashes. The agent is provided with a sufficient amount of time to freely explore its accessible environment. In a second phase of the task the environment is modified adding a visible reward (e.g., food) inside one box: to access the reward, the agent is required to recall the learned association and to perform the correct action causing the opening of the box. The task, which resembles the response pre-conditioning driven by neutral stimuli described by Reed et al. (1996), has already been solved using an early version of the three-looped model (Baldassarre et al., 2012): a comparison between the two versions of the model is provided in section 4.

## 3. Results

### 3.1. Input discrimination: effect of DA in a single loop

To show the effects different outflows of DA have on the processes performed by the Basal Ganglia, several tests have been carried out on a three-channel loop as in Figure 1: the mean activity of pools of neurons has been simulated as in Equations (1, 2) and an arbitrary input lasting 6 min, consisting of a three-dimensional vector, has been set to reach the inner cortical layer of the cortex.

**Figure 4:**
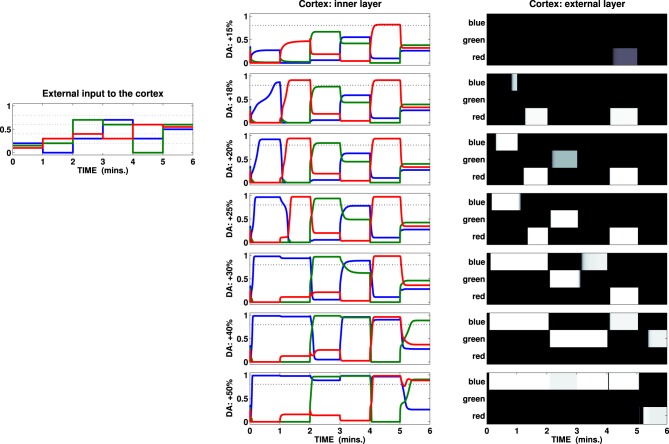
**External input (left) and activation of the channels recorded in the two cortical layers of a test striatocortical loop**. The seven different conditions (one per row, center, and right columns) are determined by the outflow of DA: center column represents activation of the three channels (three colored lines) in the inner layer of the cortex; right column represents the external layer as a B/W colormap (the channels are labeled with the same color scheme seen in the input and inner layer).

Figure 4 (left) shows values and variations of the input vector assigning a different color (blue, green, red) to each of the three-dimensions: the input changes five times during each test, with a fixed interval of 1 min. The same color code has been used to represent activation of the corresponding channels as recorded in the inner layer of the cortex and depicted in the center column of Figure 4: any of the units in the cortical inner layer may independently overcome a threshold (0.8, pictured as a dotted line) characterizing the transfer function of the external layer of the cortex. The activity in the external layer of the cortex is stabilized by the presence of lateral inhibitions preventing this layer from exhibiting multiple channel activations (Figure 4, B/W colormaps).

Given the specific set of parameters characterizing this loop, the input has been chosen so that it shows two features: first it is insufficient by itself to cause activation of any unit in the cortical external layer in the baseline DA condition. Secondly, the changes in the components in the input vector alter both sparseness (mean interval) and the overall mean value. The tests show that, depending on the amount of DA reaching the striatum, it is useful to distinguish three conditions:

1. *Weak or scarce selection*. In this condition, the striatal-cortical loop requires an input which must be both strong and sparse in order to overcome the given threshold. Therefore, few selections are performed, and—because of strong correlation with input features—they are characterized by high instability, being abandoned as soon as either the intensity of the strongest stimulus decreases or any other stimulus increases its intensity. The first row in Figure 4 (DA +15%) exemplifies this condition, showing only one activation over the threshold (fifth interval), despite the presence of stronger or equally valued stimuli in several other time intervals (i.e., third, fourth, and sixth).
2. *Enhanced discrimination*. The DA unbalances the competition between diffuse (STN) and localized (Str) signal processing, favoring the latter: this condition enables the loop to amplify the differences between stimuli with similar intensity via accumulation of the strongest signal and suppression of the weaker ones. The time required to perform this process is directly correlated with the amount of DA (within a certain range, the higher the release, the faster the amplification, and thus the selection). Despite the fact the loop is still unable to discriminate between strong, closely related stimuli (e.g., sixth time interval), this condition is shown to be the most flexible to any change in the environment allowing, in most cases, quick switches in selection depending on the values encoded in the input. In particular, the comparison between the second and the third row (DA +18% +20%) illustrates the effect of accumulation granted by the loop and its timing: a higher level of DA allows the system to reach a homeostasis characterized by values which overcome the given threshold (time intervals 1, 2, 3, and 5 result in activations in the external layer of the cortex). Each time the input is propagated back from cortex to the striatum, the higher value encoded in the input grows: comparing the first two time intervals in these rows we notice that a slight increase in the DA outflow makes the input in the first interval cause an activation roughly 30 s in advance.
3. *Maintenance and disrupted selection*. The competition between localized and diffuse activation is strongly unbalanced in favor of the former: this allows the possibility of discrimination between closely related strong inputs (e.g., sixth time interval, condition DA +40%), but at the same time it makes any selection performed persistent so causing interference and delayed switch (first to second time interval, DA +25%), maintenance (first to second time interval, DA +30%) and eventually (if the DA further increases) multiple channel activation (third to sixth intervals, DA +50%). The system is now unable to respond quickly to changes in the stimuli unless they are characterized by strong values: any selection is preserved until either the DA outflow decreases or the input changes dramatically. A further increment of the DA outflow makes the maintenance effect so strong that multiple activations in the loop become more and more likely, disrupting a selection mechanism (which in the present model is preserved in the external layer only due to the action of the lateral inhibitions). Such a condition implies difficulties in adaptation to changes in the environment, but it can also be considered as the cause of a useful “focus effect” which allows the preservation of rewarding selections in the presence of noise or distractors. Indeed, the condition of maintenance may be reached both due to elevated tonic DA release and due to high frequency burst firings causing DA accumulation (Floresco et al., 2003): this phenomenon would therefore favor both the expression of incentive salience and learning processes granting the repetition of those selections that have proximally caused the increase in the DA outflow.

### 3.2. Simulation of DA-dependent vigor

A second test has been carried out involving two segregated striatocortical loops, one characterized by six channels controlling attention via oculomotor selection (assuming the simplified environment of the mechatronic board showing only six cues to focus the attention on) and a second three channeled loop simulating the selection between three arm actions: in both cases, the loops do not receive any external input but they are provided with randomly generated noise in the thalamus. Changing every step, the noise results in a “random walk” eventually triggering random selection in the cortex. The choice of the thalamus as the locus for the random walk is justified by the reasoning that this area receives information from several cortical sources: this input is abstracted with the noise used in the model. In this respect, this noise should not be interpreted as local neural noise, but rather as the neural activity reaching the thalamus from different cortical areas and capable to overcome SNr/GPi inhibition, inducing exploration (Baldassarre et al., 2012). It is necessary to focus on a target and to select one of the arm-controlling channels to start executing any motor action: the agent requires a variable time of around 2–3 s to complete any hypothetical action on any selected target, so that both attentional and manipulation selections must be maintained for a sufficient amount of time. If the agent perseverates in its selections, the action is executed again on the same target, resulting in repetitions.

**Figure 5:**
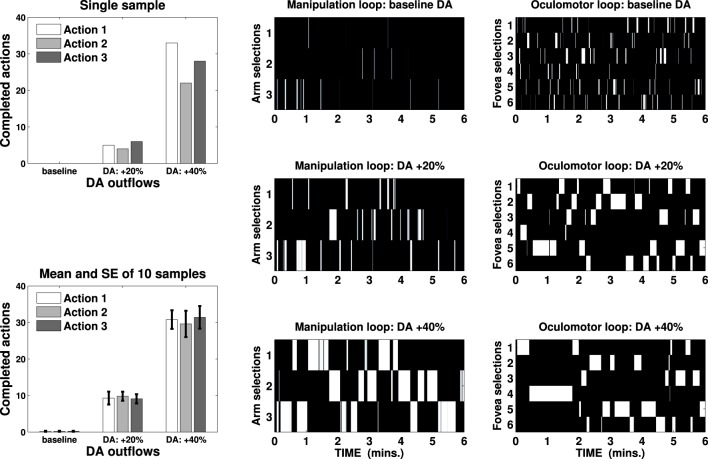
**Bin charts show completed actions in a single sample test (top) and mean and standard error of ten sample tests (bottom) in three DA conditions (baseline, +20% and +40%)**. B/W colormaps represent the neural activity of the external cortical layers of two striatocortical loops that have resulted in the action represented in the first bin chart. One loop has been used for the manipulation (three channels, center) and the other for the attentional control (six channels, right): an action is considered terminated if both the loops maintain their selection for 2 s.

We ran several tests lasting 6 min on the iCub simulator changing the DA outflow (baseline, +20% and +40%) and recording the number of completed actions performed on any possible target. The results are represented in Figure 5, which shows mean and standard error of completed actions—recorded in ten sample tests—in the three DA conditions and a sample test showing activations of external cortical layers in both the loops (B/W colormaps) and the corresponding actions performed in the three DA conditions.

These results are consistent with a known correlation between DA outflow and reward-related vigor (Niv et al., 2007; Beierholm et al., 2013) or incentive salience (Berridge and Robinson, 1998; Peciña et al., 2003), but they provide a new explanation of these behaviors. The number of completed actions increases significantly from an average of 0.6 (baseline) to 28.2 (DA +20%), reaching 91.8 (DA +40%) per test. This result is neither caused by any learning process nor it relies solely on the strengthening of the input due to DA multiplicative effect (e.g., Gurney et al., 2001b; Humphries et al., 2006): DA alters the gain of the loop thereby unbalancing the competition between the striatum and the STN, causing quick accumulation and selection at first (as seen in Figure 4, DA +20%) and then maintenance for a longer time (allowing repetitions, DA +40%).

These tests show that a widely known function ascribed to striatal DA can be produced by relying on a dynamic mechanism which allows the system to focus on a single rewarded selection as long as it is the cause of an increase of DA: the mechatronic board task exemplifies how this phenomenon both coexists and assists the standard computational role ascribed to DA as the trigger for learning processes.

### 3.3. The solution to the mechatronic board task

To solve the task the three-looped model (see Figure 2) relies on the hypothesis that, due to a differential sensitivity in the striatum, the same DA outflow causes the manipulation loop to express the first behavior (weak or scarce selection) whereas the attentional loop expresses the second (enhanced discrimination). This is consistent with data in MPTP-induced Parkinsonian subjects associating low DA outflow with the absence of motor activity but slow oculomotor foveations (Hotson et al., 1986; Schultz et al., 1989; Hikosaka et al., 2000).

This differentiation makes the agent start a visual exploration of the environment whilst performing very few arm actions (as seen in Figure 5, B/W colormaps of baseline DA condition): as soon as a novel experienced cue is perceived then activity in the HIP triggers (via NAcc and SNr) an increase in the tonic release of DA, allowing the manipulation system to enter the condition of enhanced discrimination and forcing the attentional system to a state of maintenance (as seen in Figure 5, B/W colormaps of DA +20% condition). As a consequence, the agent starts executing on a single target several randomly selected actions: the process stops when the HIP habituates to the perceived cue, restoring the usual outflow of DA, allowing the visual exploration to start again and reducing the number of action performed.

During this visual and motor exploration, the agent eventually focusses on any of the button cues: if the action “reach/press” is randomly selected and completed whilst on this target (the agent requires the usual 2–3 s of maintenance), the box associated with the pressed button opens and the corresponding light flashes. Sudden luminance changes are then perceived by the SC causing DA bursts which have a twofold effect: first, phasic DA is itself causative in a further tonic release of DA via HIP (which is highly sensitive to DA presence) therefore forcing maintenance in both manipulation and attentional loops and causing enhanced selection in the ventral loop (e.g., Figure 7, left column, 155–185 s interval). Secondly, phasic DA allows learning processes to take place, strengthening connection weights between different cortical layers and between the PFC and the DAergic area. This latter type of learning is responsible for the emergence of the agency-related predictor which results in an inhibition of DA bursts when specific motor/attention combinations are selected. As a consequence, attention is preserved on the target (the button) and the action (reach/press) is repeated until the DA bursts disappear because of the predictor, allowing the exploratory routine to restart.

The cortico-cortical learning, on the other hand, allows associating the selection of the reach/press action in the PMC and the selection of attention on the button in the FEF with the channels activated at the same time in the PFC due to the activity of the HIP. The connections established between external PMC/FEF and internal layer of PFC are essential for the predictor, whereas the connections established between the external PFC and the internal layers of PMC and FEF are essential for the agent to express goal-oriented behaviors.

**Figure 6:**
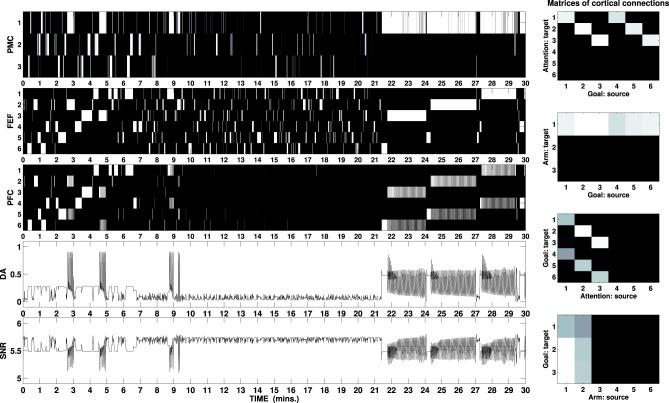
**B/W colormap represents the neural activity recorded in the external cortical layers of the three-looped neural model, labeled as PMC, FEF, and PFC (to download graphs showing the neural activity of all layers, see the Supplementary Material)**. The two line charts respectively represent the activity of the unit responsible for the simulated DA release and the sum of the activity in the SNr as part of the ventral loop. The graphs at the right represent B/W colormaps of the matrices of the cortico-cortical weights acquired during the task in the same simulation (see Supplementary Material for a video of the robot).

Figure 6 shows activity of the external cortices during a simulated task lasting 30 min. The picture outlines the first exploratory phase lasting circa 10 min: the DA outflow is increased each time a new cue is perceived and maintenance is entrained by the DA bursts when the correct motor/attention combination is found. The second phase (10–21 min) shows visual exploration and scarce arm activity: the mechatronic board has been widely explored and the cues are no longer able to elicit strong activity in the HIP.

At the beginning of the test phase, a reward becomes visible in one of the boxes. When this is detected, the high release of DA (via Amg) would make the loop maintain the wrong selection (attention on the box and any randomly selected arm-action at the time the reward is perceived): this problem is offset by the fact that the ventral loop is also activated, due to the combined effect of the high DA and the renewed activity in the HIP which also responds to high DA release. Due to the cortico-cortical connections established during the exploration phase, activity in the PFC plays the role that, in the single loop model, was ascribed to the external input. Provided the weights are strong enough, the PFC activity is then able to bias the selection in both other loops. Figure 6 shows this process of the PFC biasing the selections each time the reward is moved from one box to another (21, 24, and 27 min): within a few seconds after the reward is perceived, PFC makes the manipulation loop switch to the reach/press channel and the attentional loop switch to the selection of the button associated with the box containing the reward. Attentional loop and input reaching the NAcc are strongly connected (due to the activity in the HIP) so that when the first switches toward the button, the ventral system receives an input related to this new focus. If the correct button is pressed, the focus changes back on the box containing the reward, due to the action of the SC: the input reaching the ventral system changes again and this system eventually restarts biasing the attentional loop to focus on the associated button. This closed causal chain generates an oscillation of both attention and goal between the two targets, i.e., causing a switch of goals from an intermediate one (reach/press the correct button) to the ultimate one (reach the box to secure the food).

When the reward is moved from one box to another, the release of DA decreases, allowing the start of visual exploration until the new position of the reward is detected. Provided the agent has enough time to explore the whole environment and learn all the associations, it will be then able to solve the task.

### 3.4. Intrinsic motivations and DA control

Despite the 18 combinations of possible actions on the available targets in the environment (three actions times six cues), the simulated agent usually completes the exploration of all the possible combinations and successfully learns the three associations (after repeating each of them 5–10 times) within the first 10–12 min of a trial. For a comparison, the former version of the model, which exploits a bias in favor of the reach/press action on any target cue (due to the cortico-cortical weights established during learning), took nonetheless an average of 30 min to complete the learning process (Baldassarre et al., 2012).

The behavioral advantage of the new model is evident in the embodied tests carried out on the iCub: the robot is slower than its abstract counterpart and thus needs to maintain its selections until the button is completely pushed to open the box and turn the light on. The benefits accruing from the fact that attention is preserved on a single cue are twofold: first, it allows sufficient amount of time to try several randomly generated actions thereby increasing the chances of selection and completion of a “reach/press”; secondly, it indirectly allows the agent to discriminate between cues that have been already explored and cues that are still novel. By favoring unexplored cues, the agent avoids wasting time trying actions on explored ones and focusses on those that might still allow discovery of novel interactions.

This result arises directly from the manner in which different DA outflows (either caused by intrinsic or extrinsic motivations) alter the agent behavior, narrowing the information provided by the environment. The same mechanism described for the single loop dynamics is replicated in each of the three loops involved in solving the mechatronic board task, but it is triggered by different DA outflows. It is due to this different sensitivity that different effects (e.g., “maintenance” and “weak selection”) may be experienced at the same time in two different striatocortical loops of the same agent. This differentiation allows the agent to fixate attention on novel cues at a certain DA whereas the same agent repeats those actions causing unexpected changes in the environment and fixate on the goal to pursue at a different—higher—DA outflow.

**Figure 7:**
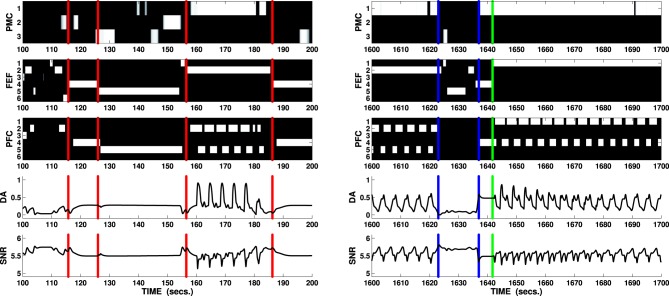
**Two sections lasting 100 s each showing key behaviors expressed by the agent solving the mechatronic board task as in Figure 6**. B/W colormaps represent the neural activity recorded in the external cortical layers of each loop (PMC, FEF, and PFC), line charts respectively represent DA release and overall neural activity in SNr (ventral loop). Vertical red lines (100–200 s interval) mark the moments when the attentional loop randomly selects a novel cue: due to the increased DA outflow (caused by the Hippocampus via NAcc and SNr), the attentional system expresses “maintenance” whereas the manipulation loop expresses enhanced exploration (e.g., third interval circa 125–155 s: the agent performs all the actions in its repertoire on the fifth cue, a box). The fourth interval (circa 155–185 s) shows the agent randomly selecting the correct action (first channel: reach/press) whilst focussing on a button (any of the first three cues): the unexpected flash of light triggers phasic DA responses causing a focussing effect in both attentional and manipulation loop and enhanced selection in the ventral loop. The predictor eventually inhibits the phasic response, allowing the system to restart its exploration routine. Vertical blue lines mark important changes in the activity of the PFC during the final test of the mechatronic board task: when the reward is relocated in a new box (1620 s: the reward is moved into the first box), the manipulation system enters a rest mode (weak selection) and the attentional system enters the exploration mode (interval between first and second blue markers 1623–1637 s). If the agent randomly selects the cue showing the reward, the DA increases reactivating the PFC (second blue marker): the ventral system is now able to bias the selections performed by both the manipulation and the attentional systems eventually forcing the switch to the proper action/target selections (green line marks the switch in the attentional loop).

The solution to the behavioral problems arising from the task can be also used to address the problem of the recorded effect DA has on the switch between model free and model-based behaviors (Wunderlich et al., 2012). During the first phase, the agent freely explores the environment guided by its random input, which resembles activity within sensorial cortices reaching the manipulation and attentional loops. This exploration can be considered as “model free” in the restricted sense that the agent does not yet have an explicit model of the environment it is exploring and it is therefore guided by the stimuli in the environment (simulated by noise). On the other hand, the more the process of learning—guided in this task by intrinsically motivating stimuli—allows establishing associations between PFC and both PMC and FEF, the more activity in PFC has the potential to bias the selection in these areas. Thus, when a reward is perceived and the PFC is activated (the ventral loop requires mid-to-high release of DA to be active), its signals guide the whole process of selection performed in both attention and manipulation loops (Figure 7, right), simulating the effect of selections guided by an acquired model of the environment and in particular of the correct combination of action on target (the button) and the resulting effect on a different cue in the environment (the box opens and the light flashes).

## 4. Discussion

The models we describe show an heterogeneous set of phenomena caused by DA affecting the working status of basal ganglia circuitry: in particular, our tests show a mechanism underlying these phenomena in the dynamic unbalancing of competition established between the direct (via D1 striatum) and hyperdirect (via STN) pathways, with high DA outflows favoring the former.

All the phenomena here simulated and tested on the iCub can be properly considered as emergent: the timing differences bringing forth vigor, maintenance causing the “focus effect” and incentive salience, the dynamic switch between behavioral strategies (rest, exploration, goal oriented behavior and model-based exploitation) do not require *ad hoc* functions or structures to be realized but instead result from intrinsic features characterizing the interaction between DA and Basal Ganglia.

The existence of segregated loops within the circuitry linking cortex and basal ganglia is currently widely accepted when considering macroscopic structures for motor, associative and limbic neural regions (Joel and Weiner, 2000; Kelly and Strick, 2004; Miyachi, 2009): within these macroscopic structures, the exact extent of the channels has been described for motor selection (Alexander and Crutcher, 1990; Romanelli et al., 2005) and the hypothesis that there are similar structures in other macroloops is consistent with findings about segregated values and saliencies within the NAcc (Samejima et al., 2005; Lau and Glimcher, 2008; FitzGerald et al., 2012).

To the best of our knowledge, the models exploiting the functions expressed by this fine grained “channeled circuitry” either investigate the effects of DA on selections performed by feed-forward models of the basal ganglia which do not close the striatocortical loop (Gurney et al., 2001b; Humphries et al., 2006, 2012), or neglect of tonic DA outflow as a regulator of the selections among distinct channels (Prescott et al., 2006; Baldassarre et al., 2012; Chersi et al., 2013).

The former type of models simulate differences in selection strength and distribution that is correlated with different DA outflows (Gurney et al., 2001b; Humphries et al., 2012), but they do not show the accumulation of signal responsible for strong alterations in selection in the presence of low and mid DA outflows. In a similar manner, the difference in the circuitry allows for an explanation of impaired switching only in terms of multiple selections (Humphries et al., 2006) but it cannot simulate the phenomena of interference and maintenance, which are described here as taking place when the DA outflow is still lower than that required for the multiple activations. Humphries et al. (2006, 2012) reach a similar conclusion about DA being responsible for an inverted U effect on the agent's ability to switch selections following changes in the environment (i.e., the external stimuli), but the differences in the neural architecture allow the present models to provide a more detailed account of the way the input is processed, especially in presence of mid-to-high DA outflows. The present models not only point out that a successful gating effect is inversely correlated with the mean value/salience ascribed to the input and directly correlated with its sparseness: the models show how, by way of the unbalanced competition, DA outflow eventually affects the spectrum of inputs that can be successfully processed by a striato-cortical loop, either increasing or decreasing it. Furthermore, the presence of the loop allows the system to maintain the performed selections making of each selection a part of the input in the following cycle, ignoring most changes in the environment. From a computational perspective, the use of a loop to create a memory-like phenomenon and preserve neural activity despite changes in the input is not novel: a similar conclusion about preserving selections (there called “latching”) has been reached for instance by Humphries and Gurney (2002). The novelty of the present study is to show how this mechanism can be caused by the dynamics of the DA outflows, hence becoming strongly correlated with the presence of motivations and rewards.

There are concerns that may be raised when establishing a comparison between the biological complexity of the neural structure of the striato-cortical loop and its simplified version implemented in our models. In particular, the results might be biased by three distinct features characterizing the architecture of the present models: first and foremost, the lack of the basal ganglia indirect pathway; second, the lack of the re-entrant cortico-thalamic loop; finally, the presence of the lateral inhibitions in the second layer of the cortex, which may perform the selections in place of the basal ganglia (as seen in Figure 4, +50% condition).

These concerns may lead to the conclusion that the simulations generated by the models are ill-grounded. However, it should be considered that the effects on selections are mainly due to the alteration of the gain obtained unbalancing the competition between direct and hyperdirect pathways due to increased release of DA, i.e., a condition paralleled and strengthened, in real brain, by a diminished activity in the indirect pathway (due to the presence of D2). Since the indirect pathway plays a major role in regulating the selections by controlling, via GPe, the activity of STN (Gurney et al., 2001a,b; Frank, 2006), the results coming from the model might have a quantitative bias in providing predictions about the amount of DA required to unbalance the competition between direct and hyperdirect pathway, but they should be sufficiently reliable in providing a general qualitative understanding on the consequences of such unbalance.

The lack of the re-entrant thalamo-cortical loop is also a possible cause of biased results. Furthermore, the present model shows a “collapsed” version of the cortical layers involved where the actual biological circuitry (Douglas and Martin, 2004; da Costa and Martin, 2010) is condensed in a single layer receiving input from the thalamus and propagating it back to the striatum, whilst a second layer is mainly used as an output source for the robotic set-up. Former models (Humphries and Gurney, 2002) have demonstrated the ability of a more complex thalamo-cortical circuitry to preserve a selection in the cortex independently of the input provided by the basal ganglia. Still, the computation performed at this level should not affect our key hypothesis about the role played by the DA in biasing the gain of the striato-cortical loop in favor of the direct pathway. From a computational perspective, the input reaching the striatum from the cortex is weighted by the presence of the DA in the area: as a result, the differences between the single values characterizing each component of this input are increased when the DA outflow increases. After this input is processed in the thalamo-cortical circuit and propagated back to the striatum, this process is repeated, so that the new cycle further increases the differences in the inputs. In the present model the computation performed in the thalamus is simplified via its basal activity, which is lowered by the inhibition provided by the SNr or GPi. A more bio-constrained model would be grounded on reciprocal connections between thalamus and cortex and these would be the cause for the initial activation of the former. We argue that this change might once again affect timing and duration of maintenance, but it would not affect the general hypothesis about the improved gain in the bigger loop involving the striatum, which is essential for the increased chance the basal ganglia have to maintain any performed selection, realizing a memory-like phenomenon. The thalamo-cortical loop is part of the striato-cortical one, so it has for sure an important effect on this maintenance, but the functions of the two structures can be considered as distinct, although affecting each other.

In future work, we plan to model an architecture of the basal ganglia including both the indirect pathway and a more complex thalamo-cortical connectivity, including the reentrant loop, though relying on the same type of computational tools and assumptions. This will allow a better comparison with the known literature via the analysis of how the functioning of this neural system is modulated when the DA release either increases above or decreases below the baseline.

Concerning the selection in the second layer of the cortex, Figure 4 shows that the mechanism of the lateral inhibition becomes important only when the DA release reaches very high values (e.g., in the single loop test, compare +50% with +20%, +30%, and five out of six intervals in the +40% condition), determining multiple selections in the first layer of the cortex. DA release recorded in most of the task is well below this threshold (see Figure 7), so that it is fair to state that the selections performed during the task are properly determined by the basal ganglia rather than by the lateral inhibition in the second layer of the cortex. An example of selections performed without the help of the lateral inhibition is provided by the ventral loop (which lacks these inhibitions in both cortical layers), where it is possible to see some overlap among selections, whilst the system is still able to perform quick switches depending on its input. It is important to stress here that the lack of lateral inhibition in the ventral system is meant mainly for the purpose to demonstrate the ability of the underlying system to perform its selections independently of the final “filter” which would be implemented in the second cortical layer. This assumption does not entail that the PFC does not have lateral inhibition, as the cortex of the other two loops do. Adding these inhibitions would have resulted in a “cleaner” output signal as the one recorded in the second layers of the attentional and manipulation loop, but it would have possibly concealed the selection of basal ganglia targeted here.

Compared to its early version (Baldassarre et al., 2012), the three-looped model has been modified mainly by altering cortico-cortical connectivity, erasing direct inputs to Cau and Put, adding the hippocampal input to the ventral loop and an agency guided predictor to stop DA bursts when a perceived stimulus is no longer unexpected. What is more important, both DA outflow dynamics and effects it plays in its target regions have been sophisticated. To solve the mechatronic board task, the model exploits the temporary focus effect, jointly with a differentiated sensitivity to DA in different striatal regions. The combination of these two features results in completely different behaviors in relation to distinct DA outflows. It is useful to distinguish three phases in the task: first, the agent visually explores the environment looking for new cues and performing few arm actions; secondly, the agent focusses attention on a new cue and randomly explores possible interactions with the cue itself thanks to its action repertoire; finally, the agent repeats those action selections responsible for generating intrinsically motivating changes in the environment or granting access to rewards.

The early version of the model also had to secure a similar behavior in presence of intrinsic motivations to boost learning processes. To this purpose, a “repetition bias” (Gurney et al., 2009; Baldassarre et al., 2012) was used in the former model. This is a transient process resembling learning and unlearning conceived to offset the well-known (in reinforcement learning field) tendency of a system to stick with the action/procedure it has learned, avoiding any subsequent exploration of the environment (a nice review of the exploration versus exploitation problem can be found in Cohen et al., 2007). This classic problem has been overcome in the present model by simply relying on the differential DA release triggered by either intrinsically (i.e., novel cues and agency-related unexpected changes in the environment) or extrinsically motivating stimuli (i.e., food): we have shown both tonic and phasic DA can be causative in selection maintenance so that even if there were no learning processes, the agent would nonetheless repeat the behavior selected when the motivating stimuli are perceived.

This mechanism, jointly with the effect DA has on accumulation and selection timing, mediates vigor-like behaviors in the agent (see Figure 5), suggesting these can be caused by the ability to quickly accumulate signals and preserve a selection rather than by biasing learning processes. The differentiation between repeated behavior and learning denotes a significant difference with respect to classic reinforcement learning models (Niv et al., 2007), but it does not entail these two phenomena do not concur in determining the agent's overall behavior. It is important to stress that the model described in Baldassarre et al. (2012) had both cortico-striatal and cortico-cortical plasticity. The present model, which aims to investigate more in depth the DA role, does not entail that cortico-striatal learning is not involved in this task. It rather points out that this learning process, though sufficient for supporting the desired behavioral changes, is not strictly necessary. The removal of such learning allows the current model to better isolate some effects of DA that are often overlooked. In particular, the present model shows that DA, aside its importance for striatal learning, has also a dynamic transient effect on striato-cortical loops, which results in a behavior resembling the one caused by learning. Any learning taking place in the striatum, though biologically plausible due to the presence of DA bursts and surely present in tasks as those considered here, would have made this dynamic effect of DA much less evident, hence was removed from the present model.

On the contrary, the mechatronic board task shows the “focus effect” enhancing both the cortical learning process during exploration and the exploitation after recalling: after the PFC has successfully biased the selections performed in the manipulation and attentional loops, the agent shows a stereotyped behavior pattern in pursuing its reward. In this context DA has still a role in helping the system to focus and maintain its selections, but the learned cortico-cortical connections trigger a switch favoring those selections that are biased by the activity in the ventral loop, rather than those that are temporally close to the increase of DA outflow. The resulting behavior shows both the features described for high vigor (short time reactions) and those characterizing incentive salience (Berridge and Robinson, 1998; Peciña et al., 2003), where the “wanting” is mediated by the stability of the activity of the ventral loop.

Since the functioning realized here is determined by the special features characterizing the neural circuitry of the striatocortical loops, our results show how manipulation, attention and executive control systems may be affected by enhanced selection, interference and maintenance, that in turn are dependent on DA outflow. The model supports the hypothesis that, in normal conditions, different types of motivating stimuli, triggering different DA outflows, modulate selection, but it also provides an interesting explanation of the dysfunctions associated with hyper activation of the D1 receptors mimicking high release of DA in any of the loops. We suggest the so-called “focus effect” in particular may provide a better explanation of the recorded behavior and neural activity associated with intrastriatal injections of amphetamine or DA agonists (Wang and Rebec, 1993; Waszczak et al., 2002; Gulley et al., 2004) or of impulsive/compulsive disorders and stereotyped behaviors in medicated Parkinson's patients (Weintraub, 2009; Djamshidian et al., 2011).

Data reported in medicated Parkinson's patients can be explained by the mechanisms we describe in terms of the underlying role of guidance by the ventral loop. The learned cortico-cortical connections represent the acquired associations between actions on specific targets and the resulting changes in the environment, so that when one of these outcomes is desired (i.e., selected in the ventral loop), the learned connections allow the ventral loop to orient the selection in the other systems, causing a switch to a goal-oriented behavior.

The low sensitivity to DA in the ventral loop means this system is only activated in presence of high—tonic or phasic—DA outflows, such as the one caused by either extrinsically or intrinsically motivating stimuli so that it is either active during learning (establishing the associations) or when exploitation is necessary to pursue a reward (consistently with Wunderlich et al., 2012). But if the agent suffers from a loss of DA release in dorsal striatum and is therefore employing DA agonists to compensate this loss, the ventral striatum (which in Parkinson's patients is usually less affected by this loss) might be activated much more frequently in contexts which are normally not connected with either extrinsic or intrinsic motivations. The more frequent selections in the ventral loop due to artificially high presence of DA—or even the fixated selection if the DA is sufficiently high—would bias any other selection either in the motor or associative loops and would therefore lead to an artificially induced hyper-incentivated salience on the perceived stimuli and therefore to compulsive behaviors.

Despite the functional analogies that can be established between the motor exploration of a biological system and its artificial simulation presented here, we note that the current implementation of the actions in the robot generates a behavior which, in both conditions of increased DA release, might lead to some misinterpretations. Indeed, both the condition expressing motor exploration of the possible interactions with a novel cue (Figure 6, 0–10 min) and the one expressing exploitation of the known associations when either intrinsic or extrinsic rewards is perceived (Figure 6, 21–30 min) might resemble a dysfunctional behavior. In particular, during motor exploration several actions are initiated and do not reach their conclusion whereas in presence of motivations the robot expresses a strongly stereotyped behavior. When analyzing these data it is important to remember that the repertoire of actions in this set-up is limited enough to grant a good test (18 possible combinations of actions on different targets) of the effect of DA in narrowing down the options and guiding exploration, but far from being close to the repertoire of actions and environment interactions that would characterize—for instance—a child or a primate when playing with the very same mechatronic board. This is of course a strong limit for the potential variety and flexibility of the final behavior. Furthermore, DA affects the maintenance of a selection performed by a system which is otherwise completely guided (in its attentional and motor selections) by the random walk set in the thalamus. Thus, it is not surprising that the actions are often initiated and then interrupted when DA outflow is not sufficient to perform a strong lock. Using random noise to initiate actions granting the autonomous exploration of an environment is a functional simplification of the real motor exploration performed by biological agents, which is likely guided by goals and constantly affected by the presence of minor intrinsic and extrinsic motivations that can be found in a rich environment. This common procedure in the field of developmental robotics (Saegusa et al., 2009; Gottlieb et al., 2013; Ivaldi et al., 2013; Moulin-Frier and Oudeyer, 2013) is sometimes called “motor babbling” and is used to overcome the need to create an otherwise infeasibly rich environment to motivate exploration.

The model described in this paper can explain a wide range of behaviors under minimal assumptions. Furthermore, it is biologically plausible—being grounded in the neuroanatomy of perceptual and action selection systems. Because the model is formulated in terms of neuronal dynamics that are associated with specific cortical and subcortical structures, it lends itself to dynamic causal modeling of empirical neuronal responses. For example, it is—in principle—possible to use Equation (1) as a model of hidden neuronal activity associated with sources of electrophysiological responses. By equipping this neuronal model with a conventional electromagnetic forward model, one can then estimate the parameters (connectivity) of the model using non-invasive EEG or MEG measurements. Crucially, one could also evaluate the Bayesian model evidence for dynamic causal models with and without dopaminergic gating or gain control implicit in Equation (1). This would nicely parallel the face validity we have established through implementation of the scheme in a neurorobotics setting.

### Conflict of interest statement

The Review Editor, Dimitris Pinotsis, declares that, despite being affiliated to the same institution as authors Vincenzo G. Fiore, Karl Friston and Raymond J. Dolan, the review process was handled objectively and no conflict of interest exists. The Review Editor, Jennifer Lewis, declares that, despite being affiliated to the same institution as author Kevin Gurney, the review process was handled objectively and no conflict of interest exists. The authors declare that the research was conducted in the absence of any commercial or financial relationships that could be construed as a potential conflict of interest.

## Footnotes

^1^http://www.icub.org/

## Supplementary material

The compiled file of the C++ libraries, the files containing all the parameters and the essential matlab code to plot graphs here described and any other recording in relation to the present simulations, plus a series of demonstrative graphs showing the neural activity in all layers of the three loops, can be downloaded here: http://www.im-clever.eu/resources/models/models/fiorewetal2013keepfocussingmodel.tar.gz

The video showing the iCub accomplishing its task can be found here: http://www.youtube.com/watch?v=vW6Gf2A3-XQ
